# Unbounded Regions of High-Order Voronoi Diagrams of Lines and Line Segments in Higher Dimensions

**DOI:** 10.1007/s00454-023-00492-2

**Published:** 2023-05-25

**Authors:** Gill Barequet, Evanthia Papadopoulou, Martin Suderland

**Affiliations:** 1 Dept. of Computer Science, The Technion – Israel Inst. of Technology, Haifa, 3200003 Israel; 2 Faculty of Informatics, Università della Svizzera italiana, 6900 Lugano, Switzerland

**Keywords:** Voronoi diagram, Lines, Line segments, Polyhedra, Higher-order, Farthest-site, Higher-dimension, Great hyperspheres, 35A01, 65L10, 65L12, 65L20, 65L70

## Abstract

We study the behavior at infinity of the farthest and the higher-order Voronoi diagram of *n* line segments or lines in a *d*-dimensional Euclidean space. The unbounded parts of these diagrams can be encoded by a *Gaussian map* on the sphere of directions $\mathbb {S}^{d-1}$. We show that the combinatorial complexity of the Gaussian map for the order-*k* Voronoi diagram of *n* line segments and lines is $O(\min \{k,n-k\}n^{d-1})$, which is tight for $n-k=O(1)$. This exactly reflects the combinatorial complexity of the unbounded features of these diagrams. All the *d*-dimensional cells of the farthest Voronoi diagram are unbounded, its $(d-1)$-skeleton is connected, and it does not have tunnels. A *d*-cell of the Voronoi diagram is called a tunnel if the set of its unbounded directions, represented as points on its Gaussian map, is not connected. In a three-dimensional space, the farthest Voronoi diagram of  $n \ge 2$ lines in general position has exactly $n(n-1)$ three-dimensional cells. The Gaussian map of the farthest Voronoi diagram of line segments and lines can be constructed in $O(n^{d-1} \alpha (n))$ time, for $d\ge 4$, while if $d=3$, the time drops to worst-case optimal $\Theta (n^2)$. We extend the obtained results to bounded polyhedra and clusters of points as sites.

## Introduction

The Voronoi diagram of a set of *n* geometric objects, called sites, is a well-known space-partitioning structure with numerous applications in diverse fields of science. The *nearest* variant partitions the underlying space into maximal regions, such that all points within one region have the same nearest site. The Euclidean Voronoi diagram of points in ${\mathbb {R}}^d$ has been thoroughly studied, see, e.g., [7, 11, 15, 21]. For non-point sites, however, it has been much less considered. The dimension *d* is assumed to be constant throughout this document.

In the plane, many algorithmic paradigms, such as plane sweep, incremental construction, and divide-and-conquer have been applied to construct the Voronoi diagram of line segments [7]. In higher-dimensional spaces, however, results are quite sparse. Already in a three-dimensional space, the algebraic description of the features, such as the edges, of the Voronoi diagram of lines become very complicated [17]. As a result, the combinatorial complexity of this diagram has been a major open problem in computational geometry [27]. There is a gap of an order of magnitude between the $\Omega (n^2)$ lower bound [4] and the only known upper bound of $O(n^{3+\varepsilon })$ [33], where *n* is the number of sites. The gap carries over (and expands) to the Voronoi diagram of lines in *d*-space, $d\ge 3$, where the known bounds are $\Omega (n^{\lfloor d/2\rfloor })$ 1 and $O(n^{d+\varepsilon })$ [33]. The lower bound is derived from *n* parallel lines whose Voronoi diagram has the same complexity as the Voronoi diagram of *n* points in $(d-1)$-dimensional space. For points in ${\mathbb {R}}^d$, the bound is $\Theta (n^{\lceil d/2\rceil })$ [7], and for $(d-2)$-dimensional hyperplanes, the lower bound is $\Omega (n^{d-1})$ [4]. To the best of our knowledge, no other lower bound, other than $\Omega (n^{\lceil {d}/2\rceil })$, is available for line segments in ${\mathbb {R}}^d$, $d>3$. Better combinatorial bounds are known only for some restricted cases [6, 12, 22, 23]. A numerically robust algorithm for computing the Voronoi diagram of lines in 3D has been given by Hemmer et al. [19].

The order-*k* (resp., farthest) Voronoi diagram of a set of sites is a partition of the underlying space into regions, such that all points in one region have the same *k* nearest sites (resp., same farthest site). Seidel [32] derived exact bounds on the maximal complexity of the Euclidean farthest Voronoi diagram of points in ${\mathbb {R}}^d$. Asymptotically, the worst-case complexity of the latter diagram remains $\Theta (n^{\lceil {d}/2\rceil })$. Edelsbrunner and Seidel [15] pointed out that the order-*k* Voronoi diagram of points in ${\mathbb {R}}^d$ can be derived from the ${\le } k$-level of an arrangement of hyperplanes in ${\mathbb {R}}^{d+1}$. Agarwal and Mulmuley provided an algorithm which computes the ${\le }k$-level of *n* hyperplanes in ${\mathbb {R}}^d$ in expected $O(n^{\lfloor {d}/2\rfloor }k^{\lfloor {d}/2\rfloor })$ time [2, 28]. For sites other than points, the problems have been mostly considered in the plane. The farthest Voronoi diagram of *n* line segments in the plane was first studied much later by Aurenhammer et al. [5], who gave results on its structure, such as it is not related to convex hulls, and an algorithm to compute it in $O(n \log n)$ time. The order-*k* counterpart of this diagram was then considered by Papadopoulou and Zavershynskyi [31], who showed that its complexity is $O(k(n-k))$, if segments are disjoint or touch at endpoints, and that it can be constructed iteratively in $O(k^2n\log n)$ time. Nevertheless, a single order-*k* Voronoi region may consist of $\Omega (n)$ disjoint faces. If segments intersect, then the number of intersections affects the complexity only if $k<n/2$ [31]. Thus, farthest and order-*k* Voronoi diagrams of segments and lines illustrate fundamental differences from their counterparts of points, such as no relation to convex hulls, and disconnected Voronoi regions. Naturally, these differences carry over to higher dimensions, which is the subject of study in this paper.

In three dimensions, the Euclidean farthest-site Voronoi diagram of lines or line segments has the property that all its three-dimensional cells are unbounded [9]. Barequet and Papadopoulou [9] used a structure on the sphere of directions, called the Gaussian map, which reflects the directions under which the cells of this diagram are unbounded. The Gaussian map essentially replaces the role of the convex hull (resp. *k*-sets) in characterizing the unbounded regions of the farthest (resp. higher-order) Voronoi diagram of non-point sites. It was first used to characterise the unbounded regions of the Hausdorff Voronoi diagram of point clusters in the plane [30].

In this paper, we study the unbounded features of order-*k* and farthest Voronoi diagrams of *n* line segments and lines in ${\mathbb {R}}^d$, by considering their Gaussian maps, and characterizing the unbounded directions of the cells in these diagrams. We derive the bound $O(\min \{k, n-k\} n^{d-1})$ on the complexity of the Gaussian map of order-*k* Voronoi diagrams for these sites. The same upper bound is implied for the number of the unbounded features of these diagrams. For the farthest-site diagram ($k=n-1$), this is $O(n^{d-1})$. For segments as sites, we prove that the complexity of the Gaussian map is $\Omega (k^{d-1})$, which is tight when $n-k = O(1)$. The complexity bound is derived from the number of vertices on the Gaussian map. This leads to a lower bound of $\Omega (k^{d-1})$ on the complexity of the entire order-*k* Voronoi diagram for line segments. For the farthest-site Voronoi diagram, this bound becomes $\Omega (n^{d-1})$, which also holds for lines as sites. As a byproduct, we derive a bound on the complexity of the arrangement of *n* great hyperspheres on ${\mathbb {S}}^{d-1}$.

Further, we describe a transformation that maps a set of lines to a set of segments, such that the two respective Gaussian maps of order-*k* Voronoi diagrams are identical. This transformation can be used to carry lower bounds from lines to segments and upper bounds from segments to lines. Table 1 summarizes most of the complexity results derived in this paper. To further summarize, all the *d*-dimensional cells of the farthest Voronoi diagram of both lines and segments are unbounded, its $(d-1)$-skeleton is connected, and it does not have tunnels. In three dimensions, the farthest Voronoi diagram of lines in general position has exactly $n(n-1)$ many 3-dimensional cells, when $n\ge 2$. We show that we can compute the Gaussian map of this diagram in $O(n^{d-1}\alpha (n))$ time by using the algorithm of Edelsbunner et al. [14], which extends to higher dimensions [3, 18], for computing the envelope of piecewise-linear functions in ${\mathbb {R}}^d$. In fact, we conjecture that this bound can be improved to $O(n^{d-1})$. In three dimensions, we can compute the Gaussian map of the farthest Voronoi diagram of lines or segments in $O(n^2)$ time, following [14], which is optimal in the worst-case. If instead bounded polyhedra of total *n* vertices, or clusters of *n* total points, are considered as sites, then the bound on the running time slightly increases to $O(n^2 \alpha (n))$.

The paper is organized as follows. In Sect. 2 we give an introduction on the basic concepts used in this paper. In Sect. 3 we describe some basic properties of farthest and order-*k* Voronoi diagrams. Section 4 studies the Gaussian map of the order-*k* Voronoi diagram for a set of line segments as sites, including bounds on its complexity and also a worst-case optimal time algorithm. In Sect. 5 the close relation to lines as sites is discussed. Section 6 looks at combining segments and lines and finally Sect. 7 concludes with generalizations to bounded polyhedra or clusters of points as sites.

**Table 1:** Worst-case complexities of structures induced by a set $E$ of *n* lines or segments in ${\mathbb {R}}^d$

Structure	Lower bound	Upper bound
${{\,\textrm{GM}\,}}({{\,\textrm{VD}\,}}_{k}(E))$	$\Omega (k^{d-1})$*	$O(\min \{k,n-k\}n^{d-1})$
${{\,\textrm{GM}\,}}(\textrm{FVD}(E))$	$\Omega (n^{d-1})$	$O(n^{d-1})$
${{\,\textrm{VD}\,}}_{k}(E)$	$\Omega (k^{d-1})$*	$O(\min \{k,n-k\}n^{d+\varepsilon })$
$\textrm{FVD}(E)$	$\Omega (n^{d-1})$	$O(n^{d+\varepsilon })$

^∗^Only for segments

## Preliminaries

### Order-*k* Voronoi Diagrams

Let $E$ be a set of sites in ${\mathbb {R}}^d$. In this paper, we consider as sites *n* (possibly intersecting) line segments or *n* lines in ${\mathbb {R}}^d$. The dimension *d* is considered constant. We denote by *d*(*x*, *y*) the Euclidean distance between two points $x,y\in {\mathbb {R}}^d$. The distance $d(x,e)$ from a point $x \in {\mathbb {R}}^d$ to a site $e\in E$ is defined as

$$\begin{aligned} d(x,e) = \min {\{d(x,y)\mid y \in e\}}. \end{aligned}$$

#### Definition 2.1

For a subset of sites $H \subset E$ of cardinality $|H| = k$, the *order-k region* of *H* is the set of points in ${\mathbb {R}}^d$ whose distance to any site in *H* is smaller than to any site not in *H*. It is denoted as

$$\begin{aligned} {{\,\textrm{reg}\,}}(H) = \{p \in {\mathbb {R}}^d\mid \forall \, h \in H \;\forall \, e\in E\setminus H:d(p,h) \le d(p,e)\}. \end{aligned}$$

**Fig. 1:**
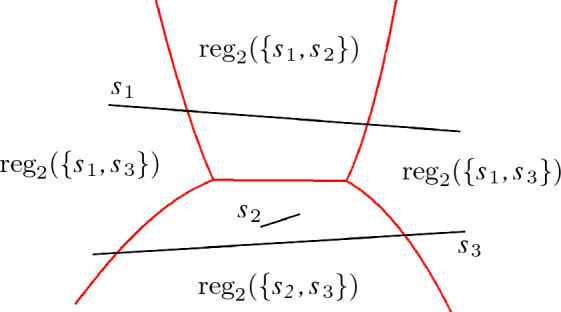
The order-2 Voronoi diagram (in red) of three segments $s_1, s_2, s_3$ in the plane

The order-*k* regions of $E$ induce a subdivision in ${\mathbb {R}}^d$. The induced cell complex is called the *order-k Voronoi diagram* of $E$, denoted by ${{\,\textrm{VD}\,}}_{k}(E)$. A maximally connected *i*-dimensional set of points, which is on the boundary of the same set of order-*k* regions, is called an *i*-dimensional cell of the cell complex. We call the *i*-dimensional cells of the order-*k* Voronoi diagram “*i*-cells.”

When $k=1$, this diagram is the well-known nearest-neighbor Voronoi diagram, denoted by ${{\,\textrm{VD}\,}}(E)$. For $k=n-1$, it is the *farthest site Voronoi diagram*, denoted by $\textrm{FVD}(E)$. Its *farthest regions* of a site $h\in E$ can also be defined directly as

$$\begin{aligned} \textrm{freg}(h) = \{p \in {\mathbb {R}}^d\mid e\in E\setminus \{h\}:d(p,h) \ge d(p,e)\}. \end{aligned}$$

### Point-Hyperplane Duality

Under the well-known point-hyperplane duality *T* in ${\mathbb {R}}^d$, a point $p \in {\mathbb {R}}^d$ is transformed to a non-vertical hyperplane *T*(*p*), and vice versa. The transformation maps a point with coordinates $(p_1,p_2,\ldots ,p_d)$ to the hyperplane *T*(*p*) satisfying the equation $x_d=-p_d+\sum _{i = 1}^{d-1} p_i x_i$. The transformation is an involution, i.e., $T = T^{-1}$.

For a segment $s=uv$, the hyperplanes *T*(*u*) and *T*(*v*) partition the dual space into four *wedges*, among which the *lower wedge* (resp., the *upper wedge*) is the one that lies below (resp., above) both *T*(*u*) and *T*(*v*). The ridge of the wedge is the intersection of *T*(*u*) and *T*(*v*).

**Fig. 2:**
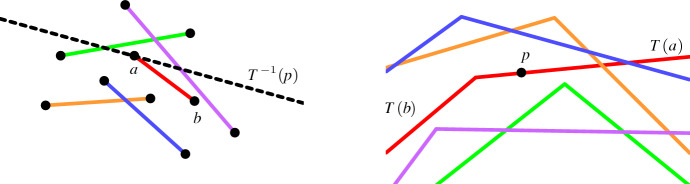
Point-hyperplane duality applied to segments: (left) Segments in primal space; and (right) their corresponding wedges in dual space

Let $E$ be a set of *n* segments, which in dual space corresponds to an arrangement of lower wedges. Let $L_k$ be the *k*th level of that arrangement. Let *p* be a point on $L_k$, which touches the dual wedge of segment $s=ab$, and let *H* be the set of segments whose wedges are below *p*, see Fig. 2. Then, the point *p* corresponds to a hyperplane $T^{-1}(p)$ which touches the segment $s$. The closed half-space above $T^{-1}(p)$ has a non-empty intersection with the segments in *H*. The open half-space above $T^{-1}(p)$ does not intersect any segment in $E\setminus H$. We will use this property when we study the Gaussian map, which is defined in the next section.

### Levels in an Arrangement of Hyperplanes

In this section, we review the definition of levels of an arrangement of surfaces, where those surfaces satisfy some *mildness* conditions (A1)–(A3) as given in [3]. We will use Theorem 2.2 by Clarkson and Shor several times in this paper.

The level of a point $p \in {\mathbb {R}}^d$ in an arrangement ${\mathcal {A}}(\Gamma )$ of a set $\Gamma$ of surface patches is the number of surfaces of $\Gamma$ lying vertically below *p*. For $0 \le k < n$, the *k*-level (resp., ${\le }k$-level), denoted by ${\mathcal {A}}_k(\Gamma )$ (resp., ${\mathcal {A}}_{{\le }k}(\Gamma )$), is the closure of all points on the surface of $\Gamma$ whose level is *k* (resp., at most *k*). A face of ${\mathcal {A}}_k(\Gamma )$ or ${\mathcal {A}}_{{\le }k}(\Gamma )$ is a maximal connected portion of a face of ${\mathcal {A}}(\Gamma )$ consisting of points having a fixed subset of surfaces lying below them. Let $\psi _k(\Gamma )$ (resp., $\psi _{{\le }k}(\Gamma )$) be the total number of faces in ${\mathcal {A}}_k(\Gamma )$ (resp., ${\mathcal {A}}_{{\le }k}(\Gamma )$) [3].

#### Theorem 2.2

(Clarkson and Shor [13])  Let ${\mathcal {G}}$ be an infinite family of surfaces satisfying some mildness assumptions (A1)–(A3) described in [3]. Then, for any $0 \le k < n-d$,

$$\begin{aligned} \psi _{{\le }k}(n,d,{\mathcal {G}}) = O\biggl ((k+1)^d\mathcal {C}\biggl (\frac{n}{k+1},d,{\mathcal {G}} \biggr )\biggr ), \end{aligned}$$

where $\mathcal {C}(n,d,{\mathcal {G}})$ is the maximum complexity of the lower envelope of *n* surfaces in ${\mathcal {G}}$.

It obviously holds that $\psi _k(\Gamma ) \le \psi _{{\le }k}(\Gamma )$.

### The Gaussian Map

Let *M* be a cell complex in ${\mathbb {R}}^d$. The *complexity* of *M* is the total number of the cells of *M* of all dimensions. The *Gaussian map* of *M* encodes information about the unbounded cells of *M*. This structure is of particular interest when all *d*-cells of *M* are unbounded. For example, all the *d*-dimensional cells of the farthest Voronoi diagram of segments or lines are unbounded.

#### Definition 2.3

A *d*-cell of *M* is *unbounded* in direction $\overrightarrow{v}$ if it contains a ray with direction $\overrightarrow{v}$.

The idea of a cell containing a ray works well for defining a cell’s unbounded directions if the cell is *d*-dimensional. However, it is not adequate for non-linear cells of dimension $<d$. For example, the trisector of three lines in 3D is in general a non-linear curve [17], containing no ray. Imagine a point *p* moving along a branch of the trisector to infinity. The tangent of the trisector at *p* does actually converge and we want to call this direction an *unbounded* direction of the trisector. The next definition refines Definition 2.3 for cells of dimension $<d$.

#### Definition 2.4

A cell *c* of *M* is *unbounded* in direction $\overrightarrow{v}$ if in the limit $\lambda \rightarrow 0$, the intersection of the scaled cell ${\lambda \cdot c}$ and the unit sphere ${\mathbb {S}}^{d-1}$ is non-empty in direction $\overrightarrow{v}$.

The scaling of cell *c* can be done with an arbitrary center. The limit $\lim _{\lambda \rightarrow 0}(\lambda c\cap {\mathbb {S}}^{d-1})$ should be understood with the concept of the Kuratowski convergence [24], which we briefly review. For any point $x \in {\mathbb {R}}^d$ and subset ${\mathcal {S}} \subset {\mathbb {R}}^d$, let $d(x,{\mathcal {S}}) = \inf {\{d(x,{\mathcal {s}}) \mid {\mathcal {s}} \in {\mathcal {S}}\}}$ be the distance between *x* and ${\mathcal {S}}$. Let ${\mathcal {S}}_\lambda \subset {\mathbb {R}}^d$ be a compact set for any $\lambda >0$. We say that ${\mathcal {S}}_\lambda$ converges to ${\mathcal {S}}$ for $\lambda \rightarrow 0$ if ${\mathcal {S}} = \{x \in {\mathbb {R}}^d \mid \limsup _{\lambda \rightarrow 0} d(x,{\mathcal {S}}_\lambda ) = 0\} = \{x \in {\mathbb {R}}^d \mid \liminf _{\lambda \rightarrow 0} d(x,{\mathcal {S}}_\lambda ) = 0\}$.

**Fig. 3:**
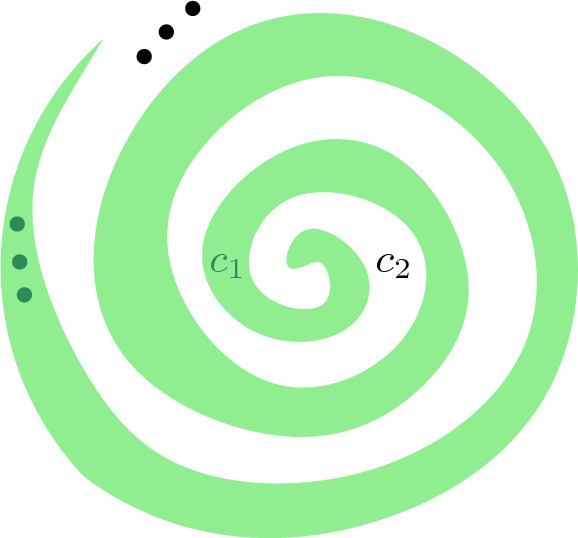
A cell complex in which none of the cells is unbounded in a specific direction

Note that the Kuratowski limit does not always exist [24]. Consider a cell complex consisting of two cells circling around each other, see Fig. 3. The unbounded directions of the cells of this cell complex are not be defined in this case, because for any cell $c \in \{c_1,c_2\}$ the sets $\{x \in {\mathbb {R}}^d \mid \limsup _{\lambda \rightarrow 0} d(x,\lambda c \cap {\mathbb {S}}^{1})=0\} = \emptyset$ and $\{x \in {\mathbb {R}}^d \mid \liminf _{\lambda \rightarrow 0} d(x,\lambda c \cap {\mathbb {S}}^{1}) = 0\} = {\mathbb {S}}^{1}$ are not the same. In this paper, we only consider cell complexes in which the unbounded directions of cells are well defined.

#### Definition 2.5

**Fig. 4:**
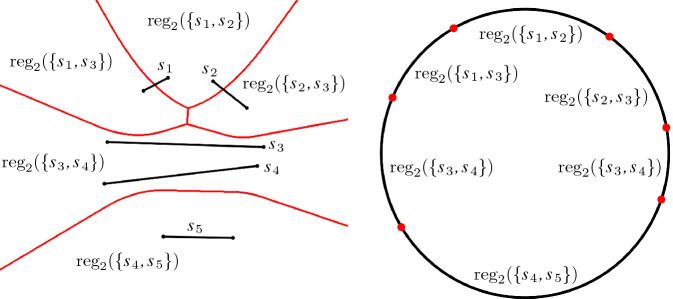
An order-2 Voronoi diagram ${{\,\textrm{VD}\,}}_{2}(\{s_1,s_2,\dots ,s_5\})$ (left) and its Gaussian map (right)

The *Gaussian map* of *M*, denoted by ${{\,\textrm{GM}\,}}(M)$, maps each cell in *M* to its unbounded directions, which are encoded on the unit sphere ${\mathbb {S}}^{d-1}$, see Fig. 4. Let *c* be a cell of *M*; the set of directions, in which *c* is unbounded, is called the *region of c* on ${{\,\textrm{GM}\,}}(M)$. The part of ${{\,\textrm{GM}\,}}(M)$ where the *d*th coordinate is $\ge 0$ (resp., $\le 0$) is called the *upper* (resp., *lower*) Gaussian map.

The Kuratowski limit is a closed set, if it exists, and therefore, cells of the Gaussian map are closed.

In this paper, we focus on cell complexes, such as the farthest Voronoi diagram and the order-*k* Voronoi diagram of lines and segments, where cells have unbounded directions and the Gaussian map is the respective partition of ${\mathbb {S}}^{d-1}$. This partition induces a cell complex on ${\mathbb {S}}^{d-1}$. The collection of cells on the Gaussian map of a Voronoi diagram ${{\,\textrm{VD}\,}}_{k}(E)$, which correspond to the same set of sites $H\subset E$, is called the *region of* *H* on ${{\,\textrm{GM}\,}}({{\,\textrm{VD}\,}}_{k}(E))$.

A Gaussian map region of a set of sites may consist of several $(d-1)$-cells for two reasons: A region of a set of sites of ${{\,\textrm{VD}\,}}_{k}$ may split into many *d*-cells, which all have unbounded directions on the Gaussian map. Moreover, the Gaussian map region of just one *d*-cell of ${{\,\textrm{VD}\,}}_{k}$ can consist of several $(d-1)$-cells, e.g., ${{\,\textrm{reg}\,}}(\{s_3,s_4\})$ in Fig. 4.

#### Definition 2.6

A *d*-cell of the order-*k* Voronoi diagram is called a tunnel if its set of unbounded directions, represented as points on its Gaussian map, is not connected.

In Fig. 4, the cell ${{\,\textrm{reg}\,}}(\{s_3,s_4\})$ forms a *tunnel* in ${{\,\textrm{VD}\,}}_{2}(E)$.

The Gaussian map essentially replaces the role of the convex hull in characterizing the unbounded regions in the higher-order Voronoi diagram of ${{\,\textrm{VD}\,}}_{k}(E))$, for $k>1$.

## Properties of the Farthest and Order-*k* Voronoi Diagram

### Combinatorial Properties

It has already been stated [9] that the complexity of the farthest Voronoi diagram is $O(n^{3+\varepsilon })$ by following the general bound of Sharir [33]. This bound generalizes for the order-*k* Voronoi diagram in ${\mathbb {R}}^d$.

#### Theorem 3.1

The order-*k* Voronoi diagram of segments and lines in ${\mathbb {R}}^d$ has complexity $O(\min \{k,n-k\}n^{d+\varepsilon })$.

#### Proof

Each site induces a distance function, which maps every point in ${\mathbb {R}}^d$ to its distance to that site. The general framework of Sharir [33] shows that the complexity of the 0-level (resp., $(n-1)$-level) of those distance functions is $O(n^{d+\varepsilon })$. Applying Theorem 2.2, the complexity of the ${\le }k$-level is $O(k n^{d+\varepsilon })$ and $O((n-k)n^{d+\varepsilon })$. $\square$

In Sect. 4 we will prove the following lower bounds. These bounds are meaningful when *k* is comparable to *n*.

#### Theorem 3.2

The complexity of the order-*k* Voronoi diagram of segments in ${\mathbb {R}}^d$ is $\Omega (k^{d-1})$ in the worst case. For the farthest Voronoi diagram ($k=n-1$), this lower bound is $\Omega (n^{d-1})$.

**Fig. 5:**
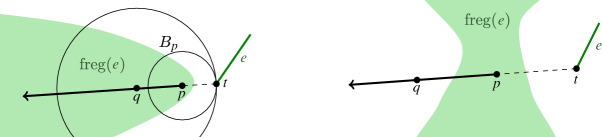
The farthest regions contain rays (left) and no farthest region can split the $(d-1)$-skeleton of the farthest Voronoi diagram into two parts (right)

### Structural Properties

#### Lemma 3.3

Let $E$ be a set of lines and segments, and let $p\in \textrm{freg}(e)$ be a point in the farthest region of site $e$. Let *t* be the point on $e$, which realizes the distance between $e$ and *p*. Then, the entire ray $\overrightarrow{r}$, that emanates from *p* with direction $\overrightarrow{tp}$, is contained in $\textrm{freg}(e)$.

#### Proof

The ball $B_p$, centered at *p* and of radius |*pt*|, touches $e$. Its interior intersects all other sites in $E$. In addition, any hyperball centered at any point $q \ne p$ along $\overrightarrow{r}$ and of radius |*qt*| must be properly enclosing $B_p$ while touching $e$ at *t*, see Fig. 5. Thus, it must also intersect all sites in $E$ except $e$. Therefore, $\textrm{freg}(e)$ must contain the entire ray $\overrightarrow{r}$. $\square$

#### Corollary 3.4

Let $E$ be a set of lines and segments. All *d*-cells of $\textrm{FVD}(E)$ are unbounded.

#### Remark 3.5

The ${{\,\textrm{VD}\,}}_{k}$ of segments can have bounded regions if $d \le k \le n-2$.

We defer the proof of Remark 3.5 to Sect. 4.

#### Definition 3.6

The *i*-skeleton of a cell complex *M* is the union of all *j*-cells in *M* with dimension ${j\le i}$.

#### Theorem 3.7

Let $E$ be a set of lines and segments in ${\mathbb {R}}^d$. The $(d-1)$-skeleton of $\textrm{FVD}(E)$ is connected.

#### Proof

Assume, for the sake of contradiction, that the diagram is not connected. Then, there exists a *d*-cell *c* that splits the $(d-1)$-skeleton into at least two parts. Let $e$ be the farthest site corresponding to *c*. The site $e$ does not touch $\textrm{freg}(e)$. Let *q* be a point, which is separated from $e$ by *c*. Let *t* be a point on $e$, which realizes the distance between *q* and $e$. Let *p* be a point on the segment ${\overline{qt}}$ in $\textrm{freg}(e)$, see Fig. 5. Then, by Lemma 3.3, the entire ray $\overrightarrow{r}$, emanating from *p* in direction $\overrightarrow{pq}$, is contained in $\textrm{freg}(e)$. In particular, $q\in \textrm{freg}(e)$, which is a contradiction. $\square$

#### Remark 3.8

The $(d-1)$-skeleton of ${{\,\textrm{VD}\,}}_{k}(E)$ need not be connected for $k \le n-2$ and $E$ being a set of sites in the plane, see Fig. 4.

## Line Segments as Sites

Let $E$ be a set of line segments in ${\mathbb {R}}^d$. We assume that the segments are in general position, i.e., no $d+1$ segment endpoints lie on the same hyperplane. First, we characterize the segments that induce unbounded regions in the order-*k* Voronoi diagram in a given direction $\overrightarrow{v}$.

### Definition 4.1

Let $E$ be a set of sites, and let *H* be a subset of $E$. A hyperplane *P* is called a *supporting hyperplane* of *H* in direction $\overrightarrow{v}$ if

- *P* is orthogonal to $\overrightarrow{v}$;
- the closed half-space $P^+$, bounded by *P* and unbounded in direction $\overrightarrow{v}$, intersects each of the sites in *H*; and
- the sites in $E\setminus H$ do not intersect the interior of $P^+$, and at least one site in $E\setminus H$ touches *P*.

**Fig. 6:**
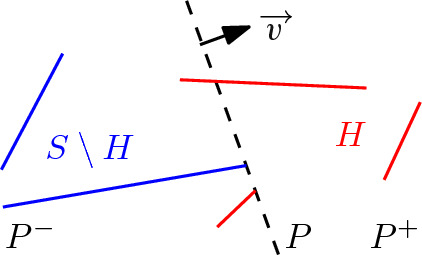
A supporting hyperplane *P* (in dashed black) of sites *H* (in red) in direction $\overrightarrow{v}$

Figure 6 illustrates a hyperplane supporting three segments.

The following theorem is a generalization of results for the plane [5, 31].

### Theorem 4.2

A set of segments *H*, with $|H | = k$, induces an unbounded region in direction $\overrightarrow{v}$ in the order-*k* Voronoi diagram of segments $S$, if and only if there exists a supporting hyperplane of *H* in direction $\overrightarrow{v}$.

### Proof

Let *H* be a set of *k* segments, which has an unbounded *d*-cell *c* in direction $\overrightarrow{v}$ in the order-*k* Voronoi diagram of a set of segments $S$. Each point in the cell corresponds to the center of a closed ball which has non-empty intersection with the segments in *H*, and does not intersect any of the other segments. By definition, there exists a curve unbounded in direction $\overrightarrow{v}$, which is contained in *c*. Any point *p* on that curve is the center of a closed ball, which has a non-empty intersection with the segments in *H* and does not intersect any of the other segments in its interior. When *p* moves along the curve to infinity, the ball around *p* becomes a half-space which is orthogonal to $\overrightarrow{v}$. By moving the bounding hyperplane in direction $-\overrightarrow{v}$ until it hits a segment in $S\setminus H$, we can make it a supporting hyperplane.

Let *P* be a supporting hyperplane of segments *H* in direction $\overrightarrow{v}$. Let $H'\subseteq H$ be the subset of segments in *H* that touch *P*. Let *x* be a point on *P*, which is closer to all endpoints of segments in $H'$ than those which belong to other segments. Consider the ray *r* which emanates from *x* and is unbounded in direction $\overrightarrow{v}$. On that ray, we find a point *y*, which is the center of a closed ball, which touches *x* and intersects only the segments in *H*. Every point *z* on *r* beyond the point *y* has the same properties because the ball keeps growing on the side $P^+$ and shrinks on the other side. This means that all those points on *r* beyond *y* belong to the order-*k* region of the set *H*. $\square$

### Corollary 4.3

A supporting hyperplane of *H* in direction $\overrightarrow{v}$, which touches *i* segments (at least one of which is in *H*), corresponds to a $(d-i+1)$-cell in ${{\,\textrm{VD}\,}}_{k}(S)$, which is unbounded in direction $\overrightarrow{v}$, and to a $(d-i)$-cell in ${{\,\textrm{GM}\,}}({{\,\textrm{VD}\,}}_{k}(S))$.

### Proof

The hyperplane is supporting several sets of sites of cardinality *k* at the same time. The farthest regions of all those sets are unbounded in direction $\overrightarrow{v}$. Moreover, these regions are adjacent to the locus of points that are equidistant from those *i* sites touching the hyperplane. This locus of points is $(d-i+1)$-dimensional and is unbounded in direction *v*, because all its adjacent regions are unbounded in *v*.

In order to work out the dimension of the Gaussian map cell in direction $\overrightarrow{v}$, we need to analyze the directions in which we can rotate the hyperplane *P*, such that it will still support the exact same set of sites. In particular, the sites which touch the hyperplane originally need to keep touching it during the rotation. If the hyperplane were touching only one endpoint of a segment, then the rotation would have $d-1$ degrees of freedom. With each additional endpoint, which touches the hyperplane, the number of degrees of freedom reduces by 1. $\square$

Note that Corollary 4.3 and its proof generalize to convex polyhedra and clusters of points as sites, see Sect. 7. We can now provide a proof deferred from Sect. 3.2.

### Proof of Remark 3.5

**Fig. 7:**
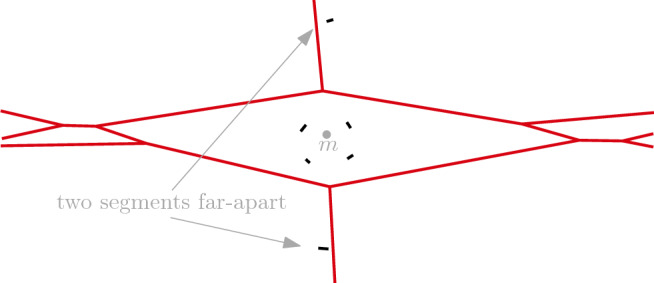
A bounded region in the order-4 Voronoi diagram (in red) of segments (in black)

Indeed, we can provide a construction for points, which can easily be extended to segments if we replace the points by small-enough segments. Put *k* points close to each other, so that there exists a point *m* in the interior of their convex hull. Add two more far-apart points, such that *m* is their midpoint, see Fig. 7. Then, the *k* points have a *d*-cell in the order-*k* Voronoi diagram. On the other hand, those *k* points do not admit a supporting hyperplane and, therefore, cannot have an unbounded *d*-cell by Theorem 4.2. It is possible to add additional points to this configuration, as long as their distance to the first *k* points is big enough. $\square$

### Theorem 4.4

Let $S$ be a set of segments. Then, $\textrm{FVD}(S)$ does not have tunnels.

**Fig. 8:**
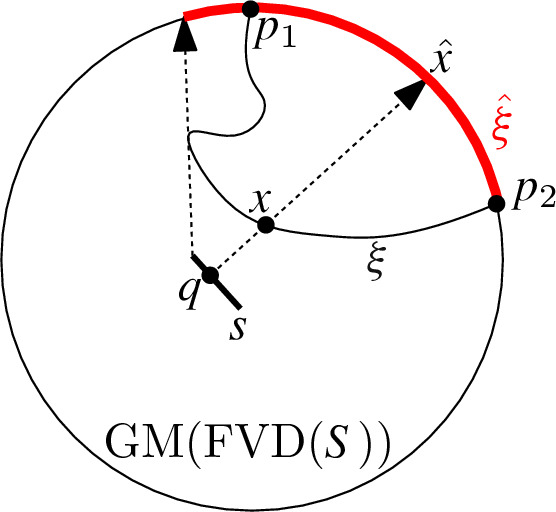
Construction of the path $\hat{\xi }$

### Proof

Let $p_1, p_2 \in {{\,\textrm{GM}\,}}(\textrm{FVD}(S))$ be two points representing unbounded directions of a farthest cell of segment $s$. These two points represent directions $\overrightarrow{r_1},\overrightarrow{r_2}$ along which there exist points $x_1, x_2 \in \textrm{freg}(s)$, for which $\overrightarrow{r_i} =\overrightarrow{q_i x_i}$ with $i = 1,2$, where $q_i$ is the point on $s$ realizing the distance between $x_i$ and $s$. Since $x_1$ and $x_2$ are contained in the same cell of $\textrm{freg}(s)$, there exists a continuous path $\xi$ connecting the points and being fully contained in $\textrm{freg}(s)$. We can map every point $x \in \xi$ to the direction $\overrightarrow{r} = \overrightarrow{q x}$, with *q* realizing the distance between *x* and $s$. We represent direction $\overrightarrow{r}$ as a point $p\in {{\,\textrm{GM}\,}}(\textrm{FVD}(S))$. Note that *p* is contained in a farthest cell of the Gaussian map corresponding to segment $s$. By continuity, mapping the whole path $\xi$ to ${{\,\textrm{GM}\,}}(\textrm{FVD}(S))$ draws a continuous path $\hat{\xi }$ between $p_1$ and $p_2$ consisting solely of points that belong to $s$. Therefore, the points $p_1$ and $p_2$ belong to the same cell of the Gaussian map. $\square$

### Remark 4.5

The order-*k* Voronoi diagram of segments $S$ can have tunnels, for $k \le n-d$.

### Proof

**Fig. 9:**
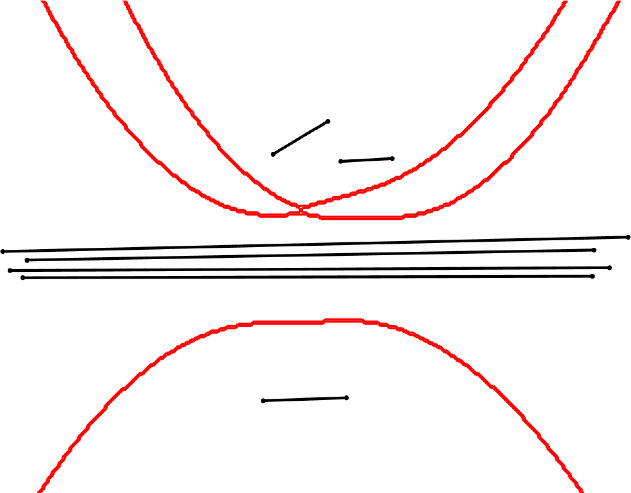
A tunnel in the order-4 Voronoi diagram (in red) of segments (in black)

Take *k* long segments very close to each other and almost parallel, and put at least *d* small segments close to and around their midpoints, see Fig. 9. Then, the order-*k* region of the *k* long segments creates a tunnel. $\square$

The next theorem provides a lower bound on the complexity of the Gaussian map of order-*k* Voronoi diagrams. This bound is meaningful if *k* is comparable to *n*.

### Theorem 4.6

Let $S$ be a set of *n* line segments in ${\mathbb {R}}^d$. A single region of the Gaussian map of the order-*k* Voronoi diagram of $S$ can have $\Omega (k^{d-1})$ many vertices.

### Proof

**Fig. 10:**
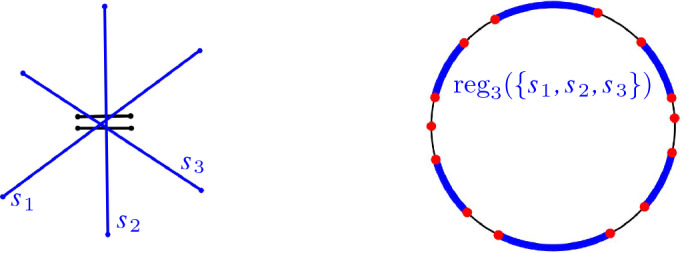
An instance of five segments (left), which has one region ${{\,\textrm{reg}\,}}(\{s_1,s_2,s_3\})$, shown in blue, on the Gaussian map of the order-3 Voronoi diagram (right) with high complexity

The bound is shown by a generalization of examples provided for the plane [5, 31]. Place *k* long segments connecting almost antipodal points on a $(d{-}1)$-di-mensional hypersphere and $n-k$ additional short segments near the center of the hypersphere, see Fig. 10. Any $(d-1)$-tuple of long segments, together with one specific short segment, define a supporting hyperplane corresponding to an unbounded edge of the order-*k* Voronoi diagram of $S$. The supporting hyperplane is spanned by an endpoint of each of the *d* segments. An unbounded edge of the diagram manifests itself as a vertex in ${{\,\textrm{GM}\,}}({{\,\textrm{VD}\,}}_{k}(S))$. All these vertices are on the boundary of the Gaussian map region of the long segments. $\square$

We can now prove Theorem 3.2.

### Proof of Theorem 3.2

Let $S$ be a set of *n* line segments in ${\mathbb {R}}^d$. In Theorem 4.6, it was stated that there can be $\Omega (k^{d-1})$ vertices in ${{\,\textrm{GM}\,}}({{\,\textrm{VD}\,}}_{k}(S))$ in the worst case. Each vertex of the Gaussian map corresponds to an edge in ${{\,\textrm{VD}\,}}_{k}(S)$. On the other hand, an edge of the diagram corresponds to at most two vertices in the Gaussian map. Therefore, the diagram contains $\Omega (k^{d-1})$ edges. $\square$

### Theorem 4.7

The complexity of the Gaussian map of the order-*k* Voronoi diagram of *n* segments in ${\mathbb {R}}^d$ is $O(\min \{k,n-k\}n^{d-1})$.

### Proof

We use the point-hyperplane duality transformation *T*, which establishes a 1-1 correspondence between the upper Gaussian map of the order-*k* Voronoi diagram and the *k*th level of the arrangement of *d*-dimensional wedges. (The lower Gaussian map is constructed in the same manner.) Each segment is mapped to a lower wedge in the dual space, which is bounded by two half-hyperplanes. Let *p* be a point in dual space. Each wedge below *p* corresponds to a segment in primal space, which has a non-empty intersection with the open half-space above $T^{-1}(p)$. Each wedge touching *p* corresponds to a segment in primal space, which is touching the closed half-space above $T^{-1}(p)$. Each wedge above *p* corresponds to a segment in primal space, whose intersection with the closed half-space above *T*(*p*) is empty. Therefore, every point on the *k*th level of the arrangement of the lower wedges corresponds to a hyperplane in primal space, which supports *k* segments. The upper or lower envelope of those *n* wedges, each composed of two half-hyperplanes, has complexity $O(n^{d-1})$ [14], recalling that we assumed the dimensions *d* to be constant. Using the bound on the lower envelope, we can now also bound the complexity of the ${\le }k$-level of the arrangement of lower wedges. We apply Theorem 2.2 to derive a complexity of

$$\begin{aligned} O\biggl ((k+1)^d \biggl (\frac{n}{k+1}\biggr )^{\!d-1}\biggr ) = O(k n^{d-1}). \end{aligned}$$

We can derive a similar upper bound of $O((n-k) n^{d-1})$ by using the complexity of the upper envelope of lower wedges as a basis. The upper Gaussian map of the order-*k* Voronoi diagram corresponds to the *k*-level of the lower wedges. Combining the two bounds completes the proof. $\square$

The bounds in Theorems 4.6 and 4.7 are tight for $n-k=O(1)$. In this case, the complexity of the Gaussian map of ${{\,\textrm{VD}\,}}_{k}$ of *n* segments is $\Theta (n^{d-1})$ in the worst case.

### Theorem 4.8

Let $S$ be a set of *n* line segments in ${\mathbb {R}}^3$. Then, ${{\,\textrm{GM}\,}}(\textrm{FVD}(S))$ can be constructed in worst-case optimal $O(n^2)$ time.

### Proof

We dualize the segments into lower wedges. The upper Gaussian map of the segments corresponds to the upper envelope of the lower wedges in dual space (recall the proof of Theorem 4.7). The upper envelope of those wedges, each composed of two halfplanes, is constructed in $O(n^2)$ time [14]. The lower Gaussian map is constructed in the same way. $\square$

The algorithm of Edelsbrunner et al. [14] for piecewise-linear functions can be extended to higher dimensions, running in $O(\alpha (n)n^{d-1})$ time [3, 18]. However, the complexity of the upper envelope of half-hyperplanes is only $O(n^{d-1})$ [14]. We suspect that the same algorithm runs in $O(n^{d-1})$ time when it computes the upper envelope of half-hyperplanes, as in ${\mathbb {R}}^3$, since the complexity of the envelope does not contain the $\alpha (n)$ factor. If so, the Gaussian map of the farthest Voronoi diagram can be constructed in $O(n^{d-1})$ time.

## Lines as Sites

Let $S$ be a set of lines in ${\mathbb {R}}^d$. We assume that the lines are in general position, i.e., the lines are non-intersecting and the directions of any *d* lines are linearly independent. In this section we derive similar conditions for the order-*k* Voronoi diagram of lines to have unbounded cells in some direction.

### Definition 5.1

**Fig. 11:**
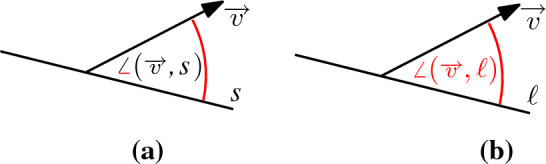
(**a**) The angular distance $\angle (\overrightarrow{v},\ell )$ between line $\ell$ and direction $\overrightarrow{v}$. (**b**) ${{\,\textrm{GM}\,}}(\textrm{FVD})$ of four lines in ${\mathbb {R}}^3$. The farthest regions of the lines are colored in different colors. Vertices of anomaly are shown with squared boxes; proper vertices with disks

For a line $\ell$ and a direction $\overrightarrow{v}$, the *angular distance* $\angle (\overrightarrow{v},\ell )$ is the smallest angle between $\overrightarrow{v}$ and the direction of $\ell$, see Fig. 11(a).

### Definition 5.2

Let $L$ be a set of lines, and let *H* be a subset of $L$. An angle $\beta$ is a *supporting angle* of *H* in direction $\overrightarrow{v}$ if

- the angular distance between $\overrightarrow{v}$ and any of the lines in *H* is at most $\beta$; and
- the angular distance between $\overrightarrow{v}$ and any of the lines in $L\setminus H$ is at least $\beta$, and at least one site in $L\setminus H$ realizes the angular distance $\beta$.

### Theorem 5.3

A set of lines *H*, with $|H | = k$, induces an unbounded region in direction $\overrightarrow{v}$ in ${{\,\textrm{VD}\,}}_{k}(L)$ if and only if *H* has a supporting angle for direction $\overrightarrow{v}$.

### Proof

Let $\ell$ be a line, and *r* be a ray emanating from an arbitrary point *p* in direction $\overrightarrow{v}$. With some calculations, one can derive that

$$\begin{aligned} \lim _{\lambda \rightarrow \infty } \frac{d(\ell ,p + \lambda \cdot \overrightarrow{v})}{\lambda } =\sin {\angle (\overrightarrow{v},\ell )}. \end{aligned}$$

The distance between the line and a point on the ray *r* increases with rate $\sin {\angle (\overrightarrow{v},\ell )}$ as the point moves along the ray to infinity. Therefore, the set of *k* lines, which minimizes the angular distance to $\overrightarrow{v}$, has an unbounded cell in direction $\overrightarrow{v}$. $\square$

### Corollary 5.4

A supporting angle of *H*, which is realized by *i* lines (at least one of which is in *H*), corresponds to an unbounded $(d-i+1)$-cell in the order-*k* Voronoi diagram of $L$.

All *d*-cells, which are unbounded in the same direction $\overrightarrow{v}$, touch at a common lower-dimensional cell. This cell is determined by the lines, which have the same angular distance to $\overrightarrow{v}$. A cell, which is equidistant to *i* lines, is $(d-i+1)$-dimensional.

### Theorem 5.5

A supporting angle $\beta$ of *H* in direction $\overrightarrow{v}$, which is realized by *i* lines (at least one of which belongs to *H*), corresponds to a $(d-i)$-cell (resp., $(d-i-1)$-cell) in ${{\,\textrm{GM}\,}}({{\,\textrm{VD}\,}}_{k}(L))$, if $\beta <\pi /2$ (resp., $\beta =\pi /2$).

### Proof

Assume first that $\beta < \pi /2$. The sites are split into three types: lines which have smaller, equal, or larger angular distance to $\overrightarrow{v}$ than the supporting angle. How can we rotate the direction $\overrightarrow{v}$ while still keeping the same partitioning of sites? In particular, having the same set of sites with the same angular distance to $\overrightarrow{v}$ as the supporting angle? If only one line had the same angular distance to $\overrightarrow{v}$, then the direction $\overrightarrow{v}$ could be rotated freely. Each additional line with the same angular distance introduces one constraint on the rotation.

Now assume that $\beta =\pi /2$. Again, we consider the partition of the lines whose angular distance to $\overrightarrow{v}$ is smaller or equal to $\pi /2$. If a line has angular distance $\pi /2$ to the direction $\overrightarrow{v}$, then this angular distance can be measured in both unbounded directions of the line. How can we rotate the direction $\overrightarrow{v}$ (that is, move the corresponding point on the sphere of directions) while still keeping the same partitioning of sites? In particular, having the same set of sites with angular distance $\pi /2$? If just one line had $\pi /2$ as angular distance, then the direction $\overrightarrow{v}$ could be rotated in $d-2$ dimensions (that is, move on the sphere with $d-2$ degrees of freedom). Each additional line with the same angular distance introduces one more constraint on the rotation, thereby reducing the degrees of freedom by one. $\square$

Typically, *i*-cells of the Gaussian map correspond to $(i+1)$-cells of the corresponding Voronoi diagram. The only exceptions are cells whose supporting angle is $\pi /2$, which correspond to $(i+2)$-cells of ${{\,\textrm{VD}\,}}_{k}$.

### Definition 5.6

The *i*-cells of the Gaussian map, $i < d-1$, which correspond to a supporting angle of $\pi /2$, are called *cells of anomaly*. All other cells are called *proper*.

In ${\mathbb {R}}^3$, the only cells of anomaly are vertices, see Fig. 11(b). Such a vertex corresponds to a direction in which the bisector of two lines seems to be self-intersecting. The bisector of two lines $\ell ,\ell '$ is a hyperbolic paraboloid. Seen “from infinity” this hyperbolic paraboloid looks like two intersecting planes. The intersection of those planes is a line *l*, which is unbounded in two antipodal directions $-\overrightarrow{v}, \overrightarrow{v}$, which are the vertices of anomaly on the Gaussian map. One of the lines $\ell ,\ell '$ is actually strictly closer to direction $\overrightarrow{v}$ than the other. Only “at infinity,” both lines seem to have equal distance in direction $\overrightarrow{v}$.

In general space ${\mathbb {R}}^d$, the *i*-cells of anomaly on the Gaussian map correspond to $(i+2)$-cells in the order-*k* Voronoi diagram. Looking at the Gaussian map, these $(i+2)$-cells seem as if they intersect, however, they do not intersect in the actual diagram. Let $\overrightarrow{v}$ be the direction of a cell of anomaly. The lines, which are orthogonal to $\overrightarrow{v}$, can actually be ordered along direction $\overrightarrow{v}$. Let *j* be the number of lines that are not orthogonal to $\overrightarrow{v}$. The region of those *j* lines, together with the closest $k-j$ orthogonal lines, is unbounded in direction $\overrightarrow{v}$ and, moreover, is not split by an $(i+1)$-cell in direction $\overrightarrow{v}$.

**Fig. 12:**
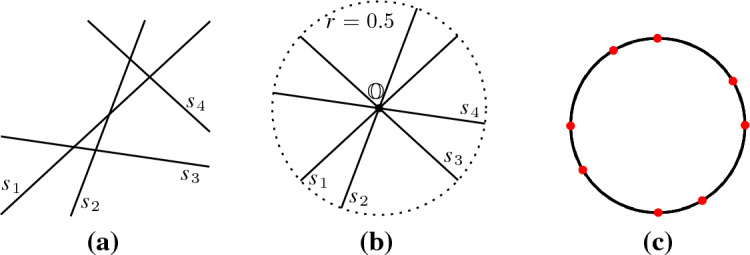
(**a**) Lines $L$ and their (**b**) transformed segments $\tau (L)$ have identical (**c**) Gaussian maps ${{\,\textrm{GM}\,}}({{\,\textrm{VD}\,}}_{2}(L))= {{\,\textrm{GM}\,}}({{\,\textrm{VD}\,}}_{2}(\tau (L)))$

We define a transformation $\tau$ that maps lines to segments. Each line $\ell$ is mapped to a unit segment $\tau (\ell )$ that has the same direction as the line and the origin ${\mathbb {O}}$ as midpoint, see Fig. 12. When applied to a set of lines, the result of the transformation is a set of segments in non-general position, but this does not affect the upper bound on the complexity of the Gaussian map.

### Theorem 5.7

Let $L$ be a set of lines. Then, ${{\,\textrm{GM}\,}}({{\,\textrm{VD}\,}}_{k}(L)) = {{\,\textrm{GM}\,}}({{\,\textrm{VD}\,}}_{k}(\tau (L)))$.

### Proof

A set of segments has an unbounded cell in direction $\overrightarrow{v}$ if and only if there exists a supporting hyperplane for those segments in direction $\overrightarrow{v}$. The supporting hyperplane *P* separates *k* segments $H \subset \tau (L)$, which have non-empty intersections with the closure of half-space $P^+$, from the other segments. The properties that the segments have unit length and the origin as midpoint guarantee that all segments in *H* have a smaller angular distance to $\overrightarrow{v}$ than any of the other segments in $\tau (L)\setminus H$. $\square$

As a consequence, lower bounds on the worst-case complexity of the Gaussian map, derived for lines as sites, carry over to segments as sites. In the same manner, all upper bounds on the worst-case complexity on the Gaussian map for segments also apply to lines. In addition, the algorithm of Theorem 4.8 to construct the Gaussian map of the farthest Voronoi diagram extends to lines as sites. (Note that the algorithm does not require the segments to be in general position.)

### Corollary 5.8

The Gaussian map of the order-*k* Voronoi diagram of *n* lines in ${\mathbb {R}}^d$ has $O(\min \{k,n-k\}n^{d-1})$ complexity. The Gaussian map can be constructed in $O(n^{d-1} \alpha (n))$ time, while if $d=3$, the time drops to $O(n^2)$.

### Theorem 5.9

Let $L$ be a set of lines. Then, $\textrm{FVD}(L)$ does not have tunnels.

For this theorem to make sense, we need to clarify our interpretation of tunnels in the presence of cells of anomaly. Recall that cells of anomaly do not correspond to any cell in the Voronoi diagram. For defining the Gaussian map, we compute the intersection of the unit sphere with the Voronoi diagram scaled by a factor $\lambda$. The Gaussian map is then derived when $\lambda$ reaches 0 in the limit. We say that a cell *c* of the Voronoi diagram forms a tunnel, if the intersection of the unit sphere with the scaled cell *c* is disconnected, for every arbitrarily small (but positive) scaling factor $\lambda$. Effectively, cells of anomaly can connect some $(d-1)$-cells of the Gaussian map, while they disconnect others.

**Fig. 13:**
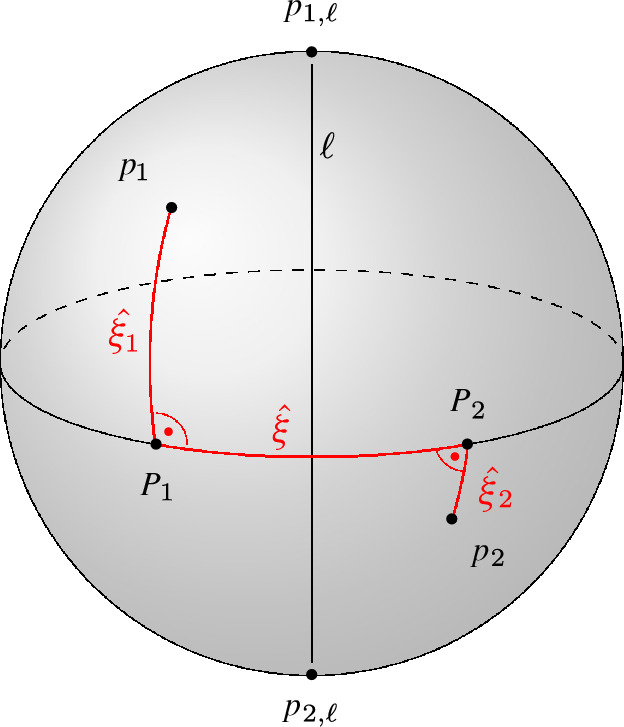
Construction of the path $(\hat{\xi _1},\hat{\xi },\hat{\xi _2})$ for lines as sites

### Proof

Let $p_1$ and $p_2$ be two points on ${{\,\textrm{GM}\,}}(\textrm{FVD}(L))$ in a farthest cell of line $\ell$. The line $\ell$ is unbounded in two directions corresponding to two points in ${{\,\textrm{GM}\,}}(\textrm{FVD}(L))$, which we call $p_{1,\ell }$ and $p_{2,\ell }$, see Fig. 13. We define the path $\hat{\xi _1} \subset {{\,\textrm{GM}\,}}(\textrm{FVD}(L))$ by starting from $p_1$ and moving along the geodesic away from the closer point of $p_{1,\ell }$ and $p_{2,\ell }$, until both points $p_{1,\ell }$ and $p_{2,\ell }$ have equal distance to our current point on $\hat{\xi _1}$. We call the last point of this path $P_1$. Similarly, we define the path $\hat{\xi _2}$ and point $P_2$ for $p_2$ as starting point. Note that by construction, the entire paths $\hat{\xi _1}$ and $\hat{\xi _2}$ contain only points corresponding to $\ell$ as the farthest line.

Now we perform the same procedure as in the proof of Theorem 4.4. The two points $P_1$ and $P_2$ represent directions $\overrightarrow{v_1}$ and $\overrightarrow{v_2}$, along which there exist points $x_1, x_2 \in \textrm{freg}(\ell )$ for which $\overrightarrow{v_i}= \overrightarrow{q_i x_i}$, with $i = 1,2$, where $q_i$ is the point on $\ell$ realizing the distance between $x_i$ and $\ell$. Since $x_1$ and $x_2$ are contained in the same cell of $\textrm{freg}(\ell )$, there exists a continuous path $\xi$ connecting both points and being fully contained in this cell. We can map every point $x \in \xi$ to the direction $\overrightarrow{v}=\overrightarrow{qx}$, with *q* realizing the distance between *x* and $\ell$, which we afterwards map to its corresponding point $p \in {{\,\textrm{GM}\,}}(\textrm{FVD}(L))$. Note that the point *p* is contained in a farthest cell corresponding to line $\ell$. By continuity, mapping the whole path $\xi$ to ${{\,\textrm{GM}\,}}(\textrm{FVD}(L))$ yields a continuous path $\hat{\xi }$ between $P_1$ and $P_2$. The path $(\hat{\xi _1},\hat{\xi },\hat{\xi _2})$ consists solely of directions in which line $\ell$ is the farthest site. $\square$

A similar construction, as in Remark 4.5, can be used for showing that ${{\,\textrm{VD}\,}}_{k}(L)$ can have tunnels for a set of lines $L$ and $k \le n-d$.

The following result stands by its own and will be used to analyze the number of *d*-cells in the farthest Voronoi diagram of lines and its Gaussian map. We look at an arrangement of great spheres with the same center and radius on a $(d-1)$-sphere. For example, consider the 2-dimensional unit sphere ${\mathbb {S}}^2$ in ${\mathbb {R}}^3$ and *n* great circles on it. We answer the following question: “Into how many 2-dimensional faces is the unit sphere split by the great circles?” We assume that no *d* great spheres have a point in common.

### Theorem 5.10

Let ${\mathbb {S}}$ be a set of *n* many $(d-2)$-dimensional unit hyperspheres in ${\mathbb {R}}^d$, centered at the origin. Then, the arrangement of ${\mathbb {S}}$ on the $(d-1)$-dimensional unit hypersphere ${\mathbb {S}}^{d-1}$ contains $$\left( {\begin{array}{c}n-1\\ d-1\end{array}}\right) + \sum _{k =0}^{d-1}\left( {\begin{array}{c}n\\ k\end{array}}\right) $$ many $(d-1)$-cells.

### Proof

**Fig. 14:**
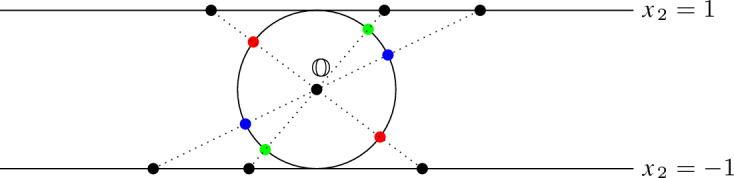
Example for $d=2$ and $n=3$: Three 0-dimensional unit spheres (blue, green, red) split the unit circle into six arcs

Each hypersphere $s\in {\mathbb {S}}$ is the intersection of ${\mathbb {S}}^{d-1}$ with a hyperplane through the origin. Therefore, the arrangement ${\mathbb {S}}$ on ${\mathbb {S}}^{d-1}$ can be seen as an arrangement of hyperplanes in ${\mathbb {R}}^d$, each containing the origin ${\mathbb {O}}$, intersected with ${\mathbb {S}}^{d-1}$. In order to compute the combinatorial structure of this intersection, we employ a bijection: a gnomonic projection of the lower and upper hemispheres of ${\mathbb {S}}^{d-1}$ (except its equator) to the two hyperplanes tangent to the poles of ${\mathbb {S}}^{d-1}$ ($x_d = -1$ and $x_d = 1$), respectively. We map every point *p* on the lower hemisphere to the intersection of the line $\overline{{\mathbb {O}} p}$ with the plane $x_d = -1$ and, similarly, every point on the upper hemisphere to the intersection of the line $\overline{{\mathbb {O}} p}$ with the plane $x_d=1$. This mapping maps the $(d-2)$-dimensional spheres in ${\mathbb {S}}$ to $(d-2)$-dimensional hyperplanes on the hyperplanes $x_d=-1$ and $x_d = 1$. See Fig. 14 for an example with $d=2$ and $n=3$. Such hyperplane arrangements have $$\sum _{k=0}^{d-1} \left( {\begin{array}{c}n\\ k\end{array}}\right) $$ many $(d-1)$-dimensional parts, among which $$\left( {\begin{array}{c}n-1\\ d-1\end{array}}\right) $$ are bounded [34]. The number of features on the hypersphere ${\mathbb {S}}^{d-1}$ is, hence, the sum of features on both hyperplane arrangements minus the number of unbounded features of one hyperplane arrangement. The unbounded features on the hyperplane arrangements are glued together on the hypersphere and, therefore, only have to be counted once. Counting the $(d-1)$-dimensional parts, we obtain the claimed count. $\square$

### Theorem 5.11

Let $L$ be a set of *n* lines. The Gaussian map of $\textrm{FVD}(L)$ has $\Theta (n^{d-1})$ many $(d-1)$-cells.

### Proof

We consider, for each line, the orthogonal directions. We get *n* many $(d{-}2)$-dimensional hyperspheres in total. Each of those hyperspheres is partitioned into $$\left( {\begin{array}{c}n-2\\ d-2\end{array}}\right) + \sum _{k=0}^{d-2} \left( {\begin{array}{c}n-1\\ k\end{array}}\right) $$ parts by the other $(n-1)$-hyperspheres due to Theorem 5.10. A direction in one of those parts is orthogonal to exactly one line in $L$ and, hence, is also part of the farthest Voronoi region of that line. In total, all *n* hyperspheres are split into $$n\bigl (\left( {\begin{array}{c}n-2\\ d-2\end{array}}\right) + \sum _{k=0}^{d-2} \left( {\begin{array}{c}n-1\\ k\end{array}}\right) \bigr )=\Theta (n^{d-1})$$ parts. Now, consider a direction $\overrightarrow{v}$ not on any hypersphere but in the farthest region of $\ell$. The shortest path on the Gaussian map from $\overrightarrow{v}$ to the hypersphere corresponding to line $\ell$ contains only directions of $\textrm{freg}(\ell )$. Therefore, there are no additional $(d-1)$-cells not containing a part of a hypersphere. $\square$

It is easy to prove that all cells of ${{\,\textrm{GM}\,}}(\textrm{FVD}(L))$ are convex, in the sense that the shortest path between any two points of a cell is contained in that cell. For a set of lines $L$ in ${\mathbb {R}}^3$, we count the number of 2-cells of ${{\,\textrm{GM}\,}}(\textrm{FVD}(L))$ and subtract the number of vertices of anomaly to derive the exact number of 3-cells in $\textrm{FVD}(L)$.

### Theorem 5.12

Let $L$ be any set of $n \ge 2$ lines in ${\mathbb {R}}^3$. Then, $\textrm{FVD}(L)$ has exactly $n^2-n$ many 3-cells.

An unbounded *i*-cell of a cell complex *M* may correspond to many $(i-1)$-cells in the Gaussian map of *M*. Therefore, we need to study carefully the Gaussian map in order to derive a lower bound on the complexity of *M*.

### Proof

We know from the proof of Theorem 5.11 that ${{\,\textrm{GM}\,}}(\textrm{FVD}(L))$ in ${\mathbb {R}}^3$ has

$$\begin{aligned} n \left( \left( {\begin{array}{c}n-2\\ 1\end{array}}\right) + \sum _{k =0}^{1} \left( {\begin{array}{c}n-1\\ k\end{array}}\right) \right) = 2 n^2 - 2 n \end{aligned}$$

2-cells. Theorem 5.9 states that each of those 2-cells of the Gaussian map corresponds to a 3-cell of the farthest Voronoi diagram, unless the 2-cells are connected through vertices of anomaly. Each vertex of anomaly is the meeting point of exactly four 2-cells of the Gaussian map. In the actual $\textrm{FVD}(L)$, two of these four 2-cells correspond to the same 3-cell. Therefore, we need to subtract the number of vertices of anomaly from the number of 2-cells on the Gaussian map to derive the number of 3-cells of the farthest Voronoi diagram. There are two vertices of anomaly for every pair of lines. Hence, the farthest Voronoi diagram has $$2 n^2 - 2 n - 2 \cdot \left( {\begin{array}{c}n\\ 2\end{array}}\right) = n^2 - n$$ many 3-cells. $\square$

### Theorem 5.13

The worst-case complexity of $\textrm{FVD}$ of *n* lines is $\Omega (n^{d-1})$.

### Proof

We bound the number of proper vertices (not those of anomaly) of ${{\,\textrm{GM}\,}}(\textrm{FVD}(L))$ from below. These vertices correspond to unbounded edges of the farthest Voronoi diagram. The set of orthogonal directions to a line is a hypersphere of dimension $d-2$ in ${{\,\textrm{GM}\,}}(\textrm{FVD}(L))$. By Theorem 5.11, the hyperspheres of all lines partition ${{\,\textrm{GM}\,}}(\textrm{FVD}(L))$ into $\Theta (n^{d-1})$ many $(d-1)$-dimensional parts. If $n\ge d$ (which is the case in the asymptotic analysis), each of those parts contains at least one proper vertex. Then, $\textrm{FVD}(L)$ has an unbounded edge in that direction. Each edge is unbounded in at most two directions. Hence, the number of edges can be bounded from below by half of the number of proper vertices of the Gaussian map. Thus, the number of edges in $\textrm{FVD}(L)$ is $\Omega (n^{d-1})$. $\square$

## Combination of Lines and Segments

Let $E=L\cup S$ be a combination of $|L| = m$ many lines and $|S|=n-m$ many line segments in $\mathbb {R}^d$. We again assume that the lines are in general position, i.e., the lines are non-intersecting and the directions of any *d* lines are linearly independent. Moreover, we assume that there is no hyperplane that contains more than *d* endpoints of sites, where lines contained in such a hyperplane are counted twice (as if the line were a segment) and otherwise are not counted at all. In this section, we describe the structure of the Gaussian map of the order-*k* Voronoi diagram of $E$, and also show how it can be computed. Using the same proof technique as Theorem 5.9, the “no-tunnel” property holds for a combination of lines and line segments as sites, i.e., a path from *p* to *q* within a cell *c* of the Voronoi diagram can be mapped to the Gaussian map.

### Observation 6.1

Let $E$ be a set of lines and segments. Then, $\textrm{FVD}(E)$ does not have tunnels.

As already mentioned before in the proof of Theorem 5.3, the distance of a line to a point, which moves along a ray to infinity, increases at a rate less than 1, if the line and ray are not orthogonal. On the other hand, the distance of a segment to such a moving point always increases at a rate of 1 in the limit. Intuitively speaking, looking at the sites from arbitrarily far along a fixed direction all the segments are further away than the lines, which are not orthogonal to that direction. Only lines that are orthogonal to the direction of consideration play the same role as segments.

### Theorem 6.2

Let $E$ be a set of lines and segments, and let $H\subseteq E$ be of cardinality *k*. The set of sites *H* induces an unbounded cell in direction $\overrightarrow{v}$ in ${{\,\textrm{VD}\,}}_{k}(E)$ if and only if either one of the following conditions holds.

- There are less than *k* lines non-orthogonal to $\overrightarrow{v}$ and there exists a supporting hyperplane in direction $\overrightarrow{v}$ of sites *H*; or
- there are at least *k* lines non-orthogonal to $\overrightarrow{v}$, the set *H* consists of lines, and there exists a supporting angle in direction $\overrightarrow{v}$ of sites *H*.

The proof of Theorem 6.2 proceeds analogously to the proof of Theorem 4.2; this is the reason for the general position assumption on the sites described in the beginning of this section.

### Definition 6.3

For a line $\ell \in L$, we define the *great sphere* $G_\ell$ to be the set of directions, which are orthogonal to $\ell$. Moreover, for $L'\subset L$ define $D^=_{L'}:=\bigcap _{\ell '\in L'} G_{\ell '} {\setminus } \bigcup _{\ell \in L{\setminus } L'}G_{\ell }$ to be the set of directions, which are orthogonal to exactly all lines in $L'$ but no other. Similarly let $D^+_{L'}:=\bigcap _{\ell '\in L'}G_{\ell '}$ be the set of directions, which are orthogonal to at least all lines in $L'$. If $L'=\emptyset$, then we let $D^=_{L'}$ and $D^+_{L'}$ be the set of all possible directions ${\mathbb {S}}^{d-1}$.

Observe that due to our general position assumption for the lines, the set $D^=_{L'}$ is empty for $|L'| \ge d$. Moreover, the sets $D^=_{L'}$ for $L' \subset L$ and $|L' | < d$ form a partition of the sphere of directions ${\mathbb {S}}^{d-1}$.

We will now describe the structure of ${{\,\textrm{GM}\,}}({{\,\textrm{VD}\,}}_{k}(E))$ within each such domain $D^=_{L'}$. For any direction $\overrightarrow{v}\in D^=_{L'}$, there are exactly $m-|L' |$ many lines non-orthogonal to it. Therefore, we derive the following relation according to Theorem 6.2:

1 $$\begin{aligned} {{\,\textrm{GM}\,}}({{\,\textrm{VD}\,}}_{k}(E))\arrowvert _{D^=_{L'}} = {\left\{ \begin{array}{ll} {{\,\textrm{GM}\,}}({{\,\textrm{VD}\,}}_{k-m+|L' |}(S\cup L'))\arrowvert _{D^=_{L'}} &{}\text {if}\, k > m-|L' |, \\ {{\,\textrm{GM}\,}}({{\,\textrm{VD}\,}}_{k}(L))\arrowvert _{D^=_{L'}} &{}\text {if }\,k \le m-|L' |. \end{array}\right. } \end{aligned}$$

Note that in the first case we slightly abuse notation in the following sense. The regions of ${{\,\textrm{GM}\,}}({{\,\textrm{VD}\,}}_{k}(E))\arrowvert _{D^=_{L'}}$ are defined by tuples of *k* many sites, whereas the regions of ${{\,\textrm{GM}\,}}({{\,\textrm{VD}\,}}_{k-m+|L' |}(S\cup L'))\arrowvert _{D^=_{L'}}$ belong to sets of sites of size $k-m$. The difference is caused by the lines $L{\setminus } L'$. With the equality symbol we want to express that the cell complexes of the Gaussian maps are the same.

We will now characterize the right-hand side of (1). More precisely, we want to bound its complexity and also give an algorithm to construct it even in a slightly bigger domain, namely $D^+_{L'}$. The domain $D^+_{L'}$ is a great sphere of dimension $d-|L' |-1$ and we will show that the Gaussian map in *d* dimensions restricted to $D^+_{L'}$ actually corresponds to a Gaussian map of sites in $d-|L' |$ dimensions. Let us formalize this intuition in the next lemmas.

### Lemma 6.4

Let $E$ be a set of lines and line segments, and let *H* be a subset of $E$. For a linear subspace *Q*, we denote by ${{\,\textrm{Proj}\,}}_Q$ the orthogonal projection to *Q*. Let *P* be a hyperplane orthogonal to *Q*. Then, *P* is a supporting hyperplane of sites *H* if and only if ${{\,\textrm{Proj}\,}}_Q(P)$ is a supporting hyperplane of sites ${{\,\textrm{Proj}\,}}_Q(H)$.

### Proof

The four properties for being a supporting hyperplane in direction $\overrightarrow{v}$ are invariant under orthogonal projection to a hyperplane which contains direction $\overrightarrow{v}$. $\square$

Let *Q* be a linear subspace and *D* its set of directions it contains. Essentially Lemma 6.4 implies, that for an arbitrary set of sites $E$ it holds that ${{\,\textrm{GM}\,}}({{\,\textrm{VD}\,}}_{k}(E))\arrowvert _{D}={{\,\textrm{GM}\,}}({{\,\textrm{VD}\,}}_{k}({{\,\textrm{Proj}\,}}_Q(E))$. Most importantly, the projection ${{\,\textrm{Proj}\,}}_Q$ of all sites can be constructed in *O*(*n*) time, and the Gaussian map in lower dimensional space can be constructed faster.

**Fig. 15:**
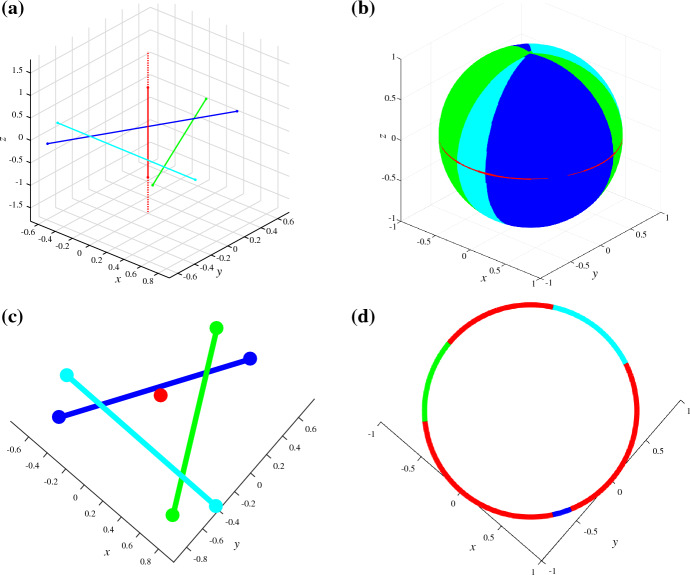
Illustration of Lemma 6.4. (**a**) The sites $E$ (one vertical red line and three line segments) in 3-space; (**b**) ${{\,\textrm{GM}\,}}(\textrm{FVD}(E))$ in $\mathbb {R}^3$; (**c**) the sites $E$ projected to the *x*-*y*-plane *Q*; (**d**) ${{\,\textrm{GM}\,}}({{\,\textrm{FVD}\,}}({{\,\textrm{Proj}\,}}_Q(E)))$ in $\mathbb {R}^2$, which corresponds to the equator of ${{\,\textrm{GM}\,}}(\textrm{FVD}(E))$

### Lemma 6.5

Let $L'$ be a subset of *L*. Then, ${{\,\textrm{GM}\,}}({{\,\textrm{VD}\,}}_{k-m+|L' |}(S\cup L'))\arrowvert _{D^+_{L'}}$ has complexity $O(\min \{k,{n-k}\} n^{d-|L' |-1})$. Further, if $k = n-1$ and $d-|L' |=3$, it can be constructed in $O(n^2)$ time.

### Proof

Let *Q* be the linear subspace, which contains exactly all directions of $D^+_{L'}$. We first project all sites $S\cup L'$ orthogonally to *Q*. Note that all lines $L'$ are orthogonal to *Q* and therefore they are projected to points. Hence ${{\,\textrm{Proj}\,}}_Q(S\cup L')$ is a set of segments, some of which degenerate to points. The space *Q* has dimension $d-|L' |$ and consequently ${{\,\textrm{GM}\,}}({{\,\textrm{FVD}\,}}({{\,\textrm{Proj}\,}}_Q(S\cup L')))$ has $O(\min \{k,n-k\}n^{d-|L' |-1})$ complexity by Theorem 4.7. If $k = n-1$ and $d-|L' |=3$, then ${{\,\textrm{GM}\,}}(\textrm{FVD}({{\,\textrm{Proj}\,}}_Q(S\cup L')))$ can be constructed in $O(n^2)$ time, see Theorem 4.8. Due to Lemma 6.4, this Gaussian map is the same as ${{\,\textrm{GM}\,}}({{\,\textrm{VD}\,}}_{k-m+|L' |}(S\cup L'))\arrowvert _{D^+_{L'}}$. $\square$

So far we have characterized the pieces of ${{\,\textrm{GM}\,}}({{\,\textrm{VD}\,}}_{k}(E))$, and it remains to put them together. A merging idea is formulated in Algorithm 1. We also use this construction to argue about the Gaussian map’s combinatorial complexity.
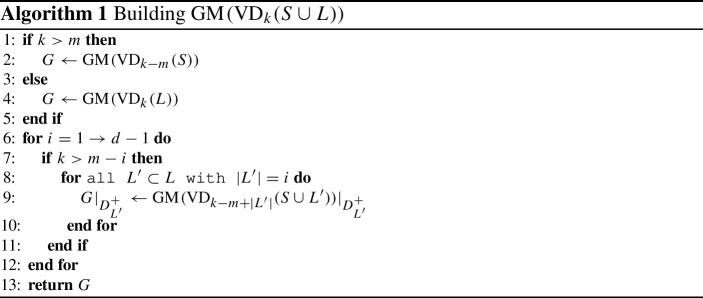


### Theorem 6.6

The complexity of the Gaussian map of the order-*k* Voronoi diagram of a combination of *n* lines and segments in ${\mathbb {R}}^d$ is $O(\min \{k,n-k\}n^{d-1})$.

### Proof

Algorithm 1 gives the outline for our complexity analysis. Depending on the relation between *k* and *m*, the initial map *G* in $\mathbb {R}^d$ is either a Gaussian map of an order-$(k-m)$ diagram of $n-m$ many sites or a Gaussian map of an order-*k* Voronoi diagram of *m* sites. In both cases the complexity can be bounded by $O(\min \{k,n-k\}n^{d-1})$ due to Theorem 4.7. After the initialization the map *G* is the correct Gaussian map in all directions, which are not orthogonal to any lines. It remains to update the map *G* along the great hyperspheres and bound the additional complexity in line 9 of Algorithm 1. Let us now consider the *i*-th update step of the for loop in line 6. We need to consider all tuples of lines of size *i*, of which there are $O(d^i)$ many. For each such set of lines $L'$, we update *G* with a Gaussian map ${{\,\textrm{GM}\,}}({{\,\textrm{VD}\,}}_{k-m+|L' |}(S\cup L'))\arrowvert _{D^+_{L'}}$, which according to Lemma 6.5 has $O(\min \{k,n-k\}n^{d-i-1})$ complexity. Updating for all such tuples of size *i* therefore increases the total complexity by $O(\min \{k,n-k\}n^{d-1})$. Since *i* runs between 1 and $d-1$, the total complexity of *G* hence does not increase asymptotically, recalling that *d* is assumed to be constant. Finally we have to address one detail, that was omitted so far. When we update *G* along a great sphere, then this sphere may actually cut features of the map in two pieces, thus also increase the complexity of the map. Let us go back to the time when *G* was updated for all tuples of lines of size $i-1$. For a fixed set of lines $L'$ of size *i*, the great sphere $D^+_{L'}$ splits some features of *G*. Consider a direction $\overrightarrow{v}$ very close to $D^+_{L'}$. The lines in $L'$ may belong to the closest *k* sites in direction $\overrightarrow{v}$ but might not do so on $D^+_{L'}$. Essentially, the number of features that are split by updating along $D^+_{L'}$ can be bounded by the complexity of ${{\,\textrm{GM}\,}}({{\,\textrm{VD}\,}}_{k-m}(S))\arrowvert _{D^+_{L'}}$, whose complexity can also be bounded from above by $O(\min \{k,n-k\}n^{d-i-1})$. Thus even the split features do not asymptotically increase the overall complexity. $\square$

Let us now look into actually constructing the Gaussian map of the farthest Voronoi diagram of lines and line segments combined in three dimensional space. But first we need to introduce the concept of a zone of great circles on a sphere similarly to the zone of a line in an arrangement of lines.

### Definition 6.7

Let ${\mathbb {S}}$ be a set of *n* many $(d-2)$-dimensional unit hyperspheres in ${\mathbb {R}}^d$, centered at the origin. These *n* hyperspheres form an arrangement ${\mathcal {A}}$ on the $(d-1)$-dimensional unit hypersphere ${\mathbb {S}}^{d-1}$. The zone of one $(d-2)$-dimensional hypersphere *s* in ${\mathcal {A}}$ is defined as the set of cells of ${\mathcal {A}}$ that bound the $d-1$ cells intersected by *s*.

### Theorem 6.8

(zone theorem for great hyperspheres)  The complexity of the zone within an arrangement of hyperspheres, as defined in Definition 6.7, is $O(n^{d-2})$.

### Proof

Similarly to Theorem 5.10, we employ the same gnomonic projection of the lower and upper hemispheres of ${\mathbb {S}}^{d-1}$ (except its equator) to the two hyperplanes tangent to the poles of ${\mathbb {S}}^{d-1}$ ($x_d = -1$ and $x_d = 1$), respectively. This projection maps the arrangement of *n* many $(d-2)$-dimensional hyperspheres in $\mathbb {R}^d$ to two arrangements of hyperplanes in $\mathbb {R}^{d-1}$. According to [16], the complexity of a zone in the arrangement of *n* hyperplanes in $\mathbb {R}^d$ is $O(n^{d-1})$. The zone within an arrangement of hyperspheres corresponds to the union of the zones in the arrangement of hyperplanes. Therefore we can also bound the complexity of the zone of hyperspheres by $O(n^{d-2})$. $\square$

### Theorem 6.9

Assume *E* is a combination of *n* lines and segments in $\mathbb {R}^3$. Then, ${{\,\textrm{GM}\,}}(\textrm{FVD}(E))$ can be constructed in worst-case optimal $O(n^2)$ time.

### Proof

We use Algorithm 1 to construct the Gaussian map. The initial map of line 2 or 4 can be constructed in $O(n^2)$ time according to Theorem 4.8. *Afterwards*, the map *G* is updated along great circles and their intersections. We need to explain the following two key steps:

- the Gaussian map along the great circles and their intersections can be computed efficiently enough; and
- merging the original map *G* with the updates along the great circles.

Regarding the first point, note that computing a Gaussian map of a farthest Voronoi diagram of segments can be computed from scratch in $O(n \log n)$ time, see [5]. Unfortunately in our case, this would not be efficient enough, because we would have to compute *m* such maps, one for each line, resulting in an $O(n^2 \log n)$ running time. In order to be more efficient, we take advantage of the already initially computed map *G*. Let $\ell \in L$ be fixed. The map *G* along the $D^=_{\ell }$ corresponds to the Gaussian map of the sites $E{\setminus }\{\ell \}$. In the update step we need to update the map such that it includes the site $\ell$. As described in Theorem 4.8, the Gaussian map corresponds to the upper envelope of lower wedges in the plane. This upper envelope has *O*(*n*) complexity and adding one wedge, corresponding to line $\ell$, can be easily done in linear time. Hence computing the ${{\,\textrm{GM}\,}}(\textrm{FVD}(E))$ along all great circles defined by $L$ takes in total $O(n^2)$ time. Finally we need to look at the $O(n^2)$ many directions, which are orthogonal to pairs of lines. Let $\ell ,\ell '\in L$ and $\overrightarrow{v}$ a direction orthogonal to both of the lines. The farthest site in direction $\overrightarrow{v}$ can be simply determined in constant time by comparing the farthest site of the initial map *G* in direction $\overrightarrow{v}$ with the two lines $\ell$ and $\ell '$. It remains to merge all the the pieces of the Gaussian map. Similarly to adding a line to an arrangement of lines in the plane, we can add great circles to an arrangement of great circles on a sphere. Due to the zone Theorem 6.8 adding one such great circle takes *O*(*n*) time, hence taking $O(n^2)$ time in total for updating the Gaussian map along all great circles. The complexity of the Gaussian map along each great circle is *O*(*n*) and therefore traversing the great circle and updating the directions which are orthogonal to two lines can also be done in linear time for each great circle, thus not changing the asymptotic running time. $\square$

## Polyhedra or Clusters of Points as Sites

Let $E$ be a set of bounded polyhedra. We assume that the sites are in general position, i.e., no $d+1$ many vertices lie on the same hyperplane. Theorem 4.2 directly generalizes for this setting.

### Theorem 7.1

A set of convex polyhedra *H*, with $|H | = k$, induces an unbounded region in direction $\overrightarrow{v}$ in the order-*k* Voronoi diagram of bounded polyhedra $E$, if and only if there exists a supporting hyperplane of *H* in direction $\overrightarrow{v}$.

Note that only extreme points of the convex hull of the site determine the site’s unbounded directions. If the sites are *n* non-crossing line segments in the plane, then the order-*k* Voronoi diagram has $O(k(n-k))$ complexity and a single order-*k* region may have *O*(*k*) many unbounded faces [31].

### Theorem 7.2

The complexity of the Gaussian map of the order-*k* Voronoi diagram of bounded polyhedra with *n* vertices in total in ${\mathbb {R}}^3$ is $O(\min \{k^2 n, (n-k)n^2\alpha (n)\})$.

### Proof

The proof idea is the same as in Theorem 4.7. We use the point-hyperplane duality transformation *T*, which establishes a 1-1 correspondence between the upper Gaussian map of the order-*k* Voronoi diagram of the polyhedra and the *k*-th level of an arrangement of polyhedra. (The lower Gaussian map is constructed in the same manner.) Each vertex of a site is mapped to a lane in the dual space. The site corresponds to the lower envelope $\mathcal {P}$ of these planes in the dual space. If a site consists of *m* points, then $\mathcal {P}$ is an unbounded convex polyhedron with at most *m* facets, which can be decomposed into *O*(*m*) many triangles.

The upper envelope of those polyhedra in dual space has $O(n^2 \alpha (n))$ complexity [14]. The lower envelope of the polyhdera in dual space has complexity *O*(*n*) [26], as it is essentially the lower envelope of *O*(*n*) hyperplanes.

Applying Theorem 2.2 we derive upper bounds on the complexity of the ${\le }k$-level of the arrangement of unbounded polyhedra:

$$\begin{aligned} O\biggl ((k+1)^3\frac{n}{k+1}\biggr )=O(k^2 n), \end{aligned}$$

which follows from the lower envelope, and

$$\begin{aligned} O\left( (n-k)^3 \left( \frac{n}{n-k}\right) ^2 \alpha \left( \frac{n}{n-k}\right) \right) = O\left( (n-k) n^2 \alpha \biggl (\frac{n}{n-k}\biggr )\right) , \end{aligned}$$

which follows from the upper envelope. The upper Gaussian map of the order-*k* Voronoi diagram corresponds to the *k*-level of the unbounded polyhedra. Combining the two bounds completes the proof. $\square$

Note that the same approach would also work to bound the complexity of the Gaussian map of the order-*k* Voronoi diagram in $d \ge 3$ dimensions, but unfortunately the bounds would become much worse. The main reason is that the decomposition of the lower envelopes $\mathcal {P}$ into simplices can have $\omega (m)$ simplices in higher dimensions.

### Theorem 7.3

Let $E$ be a set of bounded polyhedra with *n* vertices in total in ${\mathbb {R}}^3$. Then, ${{\,\textrm{GM}\,}}(\textrm{FVD}(E))$ can be constructed in $O(n^2\alpha (n))$ time.

### Proof

Recall the proof of Theorem 7.2. The lower envelope $\mathcal {P}$ of each site can be constructed in $O(n^2)$ total time [11]. Decomposing each dual polyhedron $\mathcal {P}$ into triangles can be done in time linear in its number of faces. Computing the upper envelope of these polyhedra, which is composed of *O*(*n*) many triangles, takes $O(n^2 \alpha (n))$ time [14]. This envelope corresponds to the upper Gaussian map of the sites. The lower Gaussian map is constructed in the same way. $\square$

Let a set of points in $\mathbb {R}^d$ be called a *cluster*, and let $E$ be a family of *m* such clusters of points in $\mathbb {R}^d$, where the total number of points is *n*. We assume that the sites are in general position, i.e., no $d+1$ points lie on the same hyperplane. The distance from a point $x\in \mathbb {R}^d$ to a site $e\in E$ can be measured in two different ways, depending on if the closest or farthest point in a cluster is considered.

- $$d_{\min }(x,e) = \min {\{d(x,y) \mid y \in E\}}$$,
- $$d_{\max }(x,e) = \max { \{d(x,y) \mid y \in E\}}$$.

We can define two different types of *cluster Voronoi diagrams* on $E$ depending on the distance function under consideration, $d_{\min }$ or $d_{\max }$. The nearest Voronoi diagram induced by $d_{\max }$ on $E$ has been termed the Hausdorff Voronoi diagram of $E$ and has been essentially considered in the plane, see e.g., [14, 30]. On the other hand, the farthest Voronoi diagram of $E$ using $d_{\min }$ was first considered by Huttenlocher et al. [20], and it has also been termed the *farthest color Voronoi diagram*, see e.g., [25] and references therein. Voronoi diagrams of clusters of objects as sites have been considered in [1, 8, 20, 29]. The nearest (resp., farthest) Voronoi diagram induced by $d_{\max }$ (resp., $d_{\min }$) corresponds to the Hausdorff (resp., farthest cluster) Voronoi diagram [25, 29]. For $H\subset E$, denote by ${{\,\textrm{reg}\,}}_{k\text {-}{\max }}(H)$ (resp., ${{\,\textrm{reg}\,}}_{k\text {-}{\min }}(H)$) the Voronoi regions of *H* induced by $d_{\min }$ (resp., $d_{\max }$).

### Theorem 7.4

The Gaussian map of the order-*k* Voronoi diagram of clusters as sites induced by $d_{\min }$ is the same as the Gaussian map of the order-*k* Voronoi diagram of bounded polyhedra, if the clusters consist of the vertices of the polyhedra.

### Proof

The distance to a bounded polyhedron, seen from a point at “infinity,” is realized by a vertex of the polyhedron. $\square$

Hence, the bound described in Theorem 7.2 also holds for the Gaussian map of the order-*k* Voronoi diagram of clusters as sites induced by $d_{\min }$.

### Observation 7.5

Let $E$ be a set of clusters and *H* a subset of cardinality *k*. Then, ${{\,\textrm{reg}\,}}_{k\text {-}{\min }}(H)$ is unbounded in direction $\overrightarrow{v}$ if and only if ${{\,\textrm{reg}\,}}_{k\text {-}{\max }}(E\setminus H)$ is unbounded in direction $-\overrightarrow{v}$.

In particular, the Gaussian map of the order-*k* Voronoi diagram induced by $d_{\min }$ is a reflection at the origin of the Gaussian map of the order-$(n-k)$ Voronoi diagram induced by $d_{\max }$.

## Conclusion and Open Problems

We derived bounds on the complexity of the order-*k* Voronoi diagram of lines and line segments in $\mathbb {R}^d$ and its Gaussian map, listed in Table 1. The results are tight for the farthest Voronoi diagram. We also provided an algorithm to compute the Gaussian map of the farthest Voronoi diagram in three dimensions in a worst-case optimal time. It remains an open problem to determine whether or not the lower bounds on the complexity of ${{\,\textrm{VD}\,}}_{k}$ and ${{\,\textrm{GM}\,}}({{\,\textrm{VD}\,}}_{k})$ for segments, as listed in Table 1, extend also to lines, when $k < n-1$.

There is a gap between our lower and upper bounds on the complexity of the Gaussian map of the order-*k* Voronoi diagram. What is the correct bound and how can the diagram be constructed efficiently? This question is related to [27, Prob. 3]: “What is the combinatorial complexity of the Voronoi diagram of a set of lines (or line segments) in three dimensions?”

We believe that knowing the structure of the Gaussian map, or equivalently, the structure of *d*-dimensional cells of the order-*k* Voronoi diagram, is a fundamental first step that can help in analyzing the whole diagram. It may also be useful in constructing the full diagram. We leave this question for further research.

## Footnotes

1 Boris Aronov, personal communication (2019).
